# High-Performance Piezoelectric-Type MEMS Vibration Sensor Based on LiNbO_3_ Single-Crystal Cantilever Beams

**DOI:** 10.3390/mi13020329

**Published:** 2022-02-19

**Authors:** Huifen Wei, Wenping Geng, Kaixi Bi, Tao Li, Xiangmeng Li, Xiaojun Qiao, Yikun Shi, Huiyi Zhang, Caiqin Zhao, Gang Xue, Xiujian Chou

**Affiliations:** 1 Science and Technology on Electronic Test and Measurement Laboratory, North University of China, Taiyuan 030051, China; b1806002@st.nuc.edu.cn (H.W.); bikaixi@nuc.edu.cn (K.B.); s2006010@st.nuc.edu.cn (T.L.); xiaojunqiao@nuc.edu.cn (X.Q.); s202106035@stu.nuc.edu.cn (H.Z.); s1906211@st.nuc.edu.cn (C.Z.); s1906061@st.nuc.edu.cn (G.X.); 2 Shanxi Provincial Key Laboratory of Advanced Manufacturing Technology, North University of China, Taiyuan 030051, China; xmli123@nuc.edu.cn; 3 Beijing Space Trek Technology Co., Ltd., Beijing 100176, China; shiyikun@startrekspace.cn

**Keywords:** MEMS, vibration sensor, four-cantilever beam, single-crystal LiNbO_3_, output charge sensitivity, temperature dependence

## Abstract

It is a great challenge to detect in-situ high-frequency vibration signals for extreme environment applications. A highly sensitive and robust vibration sensor is desired. Among the many piezoelectric materials, single-crystal lithium niobate (LiNbO_3_) could be a good candidate to meet the demand. In this work, a novel type of micro-electro-mechanical system (MEMS) vibration sensor based on a single crystalline LiNbO_3_ thin film is demonstrated. Firstly, the four-cantilever-beam MEMS vibration sensor was designed and optimized with the parametric method. The structural dependence on the intrinsic frequency and maximum stress was obtained. Then, the vibration sensor was fabricated using standard MEMS processes. The practical intrinsic frequency of the as-presented vibration sensor was 5.175 kHz, which was close to the calculated and simulated frequency. The dynamic performance of the vibration sensor was tested on a vibration platform after the packaging of the printed circuit board. The effect of acceleration was investigated, and it was observed that the output charge was proportional to the amplitude of the acceleration. As the loading acceleration amplitude is 10 g and the frequency is in the range of 20 to 2400 Hz, the output charge amplitude basically remains stable for the frequency range from 100 Hz to 1400 Hz, but there is a dramatic decrease around 1400 to 2200 Hz, and then it increases significantly. This should be attributed to the significant variation of the damping coefficient near 1800 Hz. Meanwhile, the effect of the temperature on the output was studied. The results show the nearly linear dependence of the output charge on the temperature. The presented MEMS vibration sensors were endowed with a high output performance, linear dependence and stable sensitivity, and could find potential applications for the detection of wide-band high-frequency vibration.

## 1. Introduction

Outer-space explorations, equipment health monitoring, the routine maintenance of machinery, and so forth, have an increasing demand for high-performance vibration sensors [1,2,3]. Compared to piezo-resistive [4] and capacitive [5] transducers, piezoelectric transducers exhibit an expected piezoelectric coupling over wireless passive sensing, a high quality factor, a large linear amplitude range and low power consumption [6,7]. Many studies have focused on piezoelectric vibration devices. Li et al. developed an asymmetric cruciform piezoelectric harvester with PZT-5H under the d_15_ mode [8]. Derakhshani et al. reported clamped–clamped and bi-stable buckled beam vibration energy harvesters with polyvinylidene fluoride (PVDF) [9]. However, piezoelectric vibration devices with large sizes cannot meet the demand for the miniaturization of sensors with high sensitivity [10]. Particularly, traditional devices have an unstable performance or even fail in extreme environments. Micro-electro-mechanical system (MEMS) piezoelectric vibration devices are considered to be one of the most sensitive among the miniaturized devices, whether measured or in design. Shen et al. reported a micromachined lead zirconate titanate (PZT) cantilever for vibration energy harvesting [11,12]. Jeon developed an interdigitated top electrode to introduce the d_33_ piezoelectric mode [13]. Ren and Zhou et al. proposed a shear mode cantilever using a single-crystal PMN-PT [14,15]. The above analyses based on a coupling bending–torsional model are helpful for the design of piezoelectric sensors. The effects of the material properties and vibration modes on the energy harvesting performance also provide some useful guidelines for the design of piezoelectric sensors.

Lithium niobate (LiNbO_3_) is a type of lead-free ferroelectric single crystal, and its piezoelectric constant is quite robust and less temperature-dependent. Islam et al. reported the measurement that the piezoelectric coefficient d_15_ of LiNbO_3_ can decrease only to about 7% even at a rather low temperature, and the coefficient is constant at a temperature below 50 K [16]. Thus, LiNbO_3_ is a better piezoelectric material candidate for many applications in extreme low-temperature environments. However, thin-film LiNbO_3_ is a kind of brittle, tough, functional, ceramic material, and it is difficult to etch into a certain shape with a relatively high-aspect-ratio structure. Qu et al. adopted a manner of focused ion beam (FIB) milling to achieve homogeneous and ultra-shallow LiNbO_3_ structures of several nanometers in thickness [17]. Ying Li et al. used a proton-exchanged wet etching technique to selectively remove a certain area of the LiNbO_3_ thin film [18]. The abovementioned methods have shown that the etching depth of LiNbO_3_ is quite shallow, only hundreds of nanometers, while the etching rate is about 10 nm per min. Xiang et al. adopted the technology of proton exchange and ion-beam-enhanced etching to fabricate ridge wave-guides with etching depths up to 2.5 μm [19]. The existing difficulties in the patterning of LiNbO_3_ thin film have hindered the application in MEMS vibration. Therefore, it is desirable to investigate LiNbO_3_-based MEMS sensors, and to validate their application in a large range of frequencies and temperatures. Fortunately, the commercially available substrate of a thin-film LiNbO_3_ bonded silicon wafer can reduce the difficulty in fabricating the sensitive structure for the MEMS sensors.

Herein, a novel MEMS vibration sensor based on functional LiNbO_3_ cantilever beams is proposed. In order to design an applicable vibration sensor, the effect of the geometric dimension on the intrinsic frequency and stress distribution on the cantilever beams was investigated in a numerical simulation. Then, the vibration sensor was fabricated using standard MEMS processes. During the fabrication, LiNbO_3_ thin-film patterning was achieved using ion-beam etching (IBE) technology. Next, the vibration sensor was packaged on a vibrating test platform in order to investigate the performance of the output charge and sensitivity under input vibration with a large range of acceleration frequencies and amplitudes. Moreover, the effect of the environmental temperature on the performance of the vibration sensor was studied.

## 2. Methods

### 2.1. Working Principle

**Figure 1:**
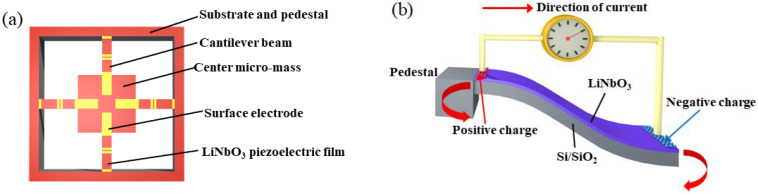
Schematic illustration of (**a**) a structural model of the vibration sensor and (**b**) the opposite electrical charges generated on different regional surfaces on the LiNbO_3_ films on the cantilever beam.

The main function of the proposed MEMS sensor is to sense vibrations with a relatively large range of frequencies in a small amplitude. The piezoelectric LiNbO_3_ layer plays an important role in response to such mechanical signals dynamically. Figure 1a schematically illustrates the MEMS vibration sensor with a LiNbO_3_ single-crystalline film bonded on silicon. The four cantilever beams are connected to a central proof mass and a square silicon frame. Meanwhile, the surface electrodes were coated on top of the LiNbO_3_ layer. With such a structural arrangement and surface electrode distribution, the d_33_ piezoelectric mode could be formed naturally. A good output performance and sensitivity can be obtained using the d_33_ mode owing to its relatively large piezoelectric coefficient [20,21]. As the mechanical vibration is applied to the central proof mass, bending deformation will occur at the cantilever beams. By this means, the deflection of the cantilever beam will lead to the piezoelectric effect of the LiNbO_3_ layer, such that the opposite potential will be formed on the surface electrode pairs.

The electrodes were set on the surface of the LiNbO_3_ layer regions with tensile stress to generate positive charges and compressive stress to obtain negative charges, as indicated in Figure 1b. By using the d_33_ mode, the positive and negative charges can be extracted from the adjacent interface by a pair of surface electrodes. The design of the electrode distribution can help to collect the generated charges from the piezoelectric layer because it is similar to the interdigital electrode, as shown in reference [20]. Although the d_33_ mode demonstrated here seems different from the classical piezoelectric modes, it is useful for our presented devices, and can obtain a higher performance.

### 2.2. Design of the Vibration Sensor

The dimensional parameters of the cantilever beam and the center mass have a direct influence on the resonant frequency and maximum stress of the vibration sensor. In order to determine the geometric dimensions of the cantilever beam and the proof mass, parametric analyses should be performed. The reason is that the practical MEMS sensor must work at a range of frequencies far away from the intrinsic frequency. Because the target range of the working frequency in practical applications is about 20 Hz to 2.4 kHz, the intrinsic frequency of the MEMS sensor should be at least 6 kHz. In this work, the LiNbO_3_ thin films and electrodes are located at the same sides, on the top surface of the cantilever beam. The four-cantilever beam sensor is geometrically symmetric in both the X and Y directions. The thickness of the sputtering-deposited gold electrodes is 200 nm; thus, it can be neglected compared to the total thickness of the cantilever beam. The intrinsic frequency of the cantilever beams was calculated using the following equation [10,11]:

(1) $$f_{n}=4*\frac{v_{n}^{2}}{2*pi}\sqrt{\frac{0.236*D_{p}*w_{c}}{{\left(l-\frac{l_{m}}{2}\right)}^{3}*\left(m_{e}+m\right)}},$$

where $D_{p}=\frac{\left[E_{L}^{2}t_{L}^{4}+E_{s}^{2}t_{s}^{4}+2E_{L}E_{S}t_{L}t_{s}\left(2t_{L}^{2}+2t_{s}^{2}+3t_{L}t_{s}\right)\right]}{12\left(E_{L}t_{L}+E_{s}t_{s}\right)}$, $m_{e}=0.236m^{\prime }w_{c}\left(l-\frac{l_{m}}{2}\right)+m^{\prime }w_{c}\frac{l_{m}}{2}$, $m^{\prime }=\rho_{L}t_{L}+\rho_{S}t_{S}$, $f_{n}$ is the *n*th mode resonant frequency, $v_{n}$ is the *n*th mode eigenvalue ($v_{1}\;\mathrm{is}\;1.875)$, $w_{c}$ is the width of the cantilever beam, l is the total length of a single cantilever beam, $l_{m}$ is the length of the proof mass, *m* is the mass of the proof mass, $D_{p}$ is a function of the Young’s moduli of the two materials $E_{L}$ (LiNbO_3_) and $E_{S}$ (Si), $m_{e}$ is the effective mass of the cantilever beam at the center of the proof mass, $m^{\prime }\;\mathrm{is}$ the mass per unit area of the cantilever beam without the proof mass, $\rho_{L}$ and $\rho_{s}$ are the densities of the piezoelectric material LiNbO_3_ and the supporting layer material Si, and $t_{L}$ and $t_{S}$ are the thicknesses of the LiNbO_3_ layer and Si layers [22,23].

As can be seen in Figure S1, COMSOL Multiphysics software was adopted to investigate the effect of varied geometric dimensions on the resonant frequency and the maximum stress. The material parameter settings are listed in Table 1. Besides the material settings, a physical setting with a Mutiphysics Interface for piezoelectric devices was used during the design of the four-cantilever beam and mass structures.

**Figure 2:**
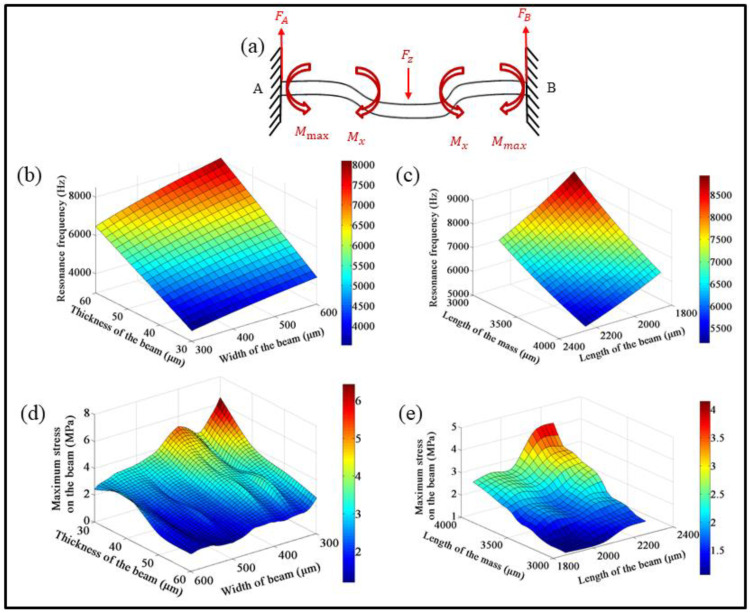
Theoretical calculation and parametric design of the structural dimensions of cantilever beams based on the resonant frequencies and maximum stress. (**a**) Schematic illustration of the force analysis and deflection of the cantilever beam under a vibration shock. (**b**) Dependence of the resonant frequency on the thickness and width of the cantilever beam. (**c**) Dependence of the resonant frequency on the lengths of the proof mass and beams. (**d**) Dependence of the maximum stress on the thickness and width of the cantilever beam. (**e**) Dependence of the maximum stress on the lengths of the proof mass and beams.

Figure 2 shows the resonant frequency and the maximum stress of the vibration sensor subjected to an external force *F_z_*. The force analysis of the cantilever beam is shown in Figure 2a. The resonant frequency and the maximum stress can be changed by varying the geometric dimensions, including the thickness and width of the cantilever beams, and the lengths of both the proof mass and the cantilever beams. In addition, a method of parametric scanning was used to analyze the effect of the structural dimension using Comsol Multiphysics. The parametric setting for the scanning analysis is listed in Table 2. The pedestal was set as a fixed constraint while the other components were set as freely moving parts.

Figure 2b,c shows that the resonant frequency would increase with the increase of the thickness and width of the cantilever beam. On the other hand, the resonant frequency would decrease continuously with the increasing of the lengths of the cantilever beam or the proof mass. Using a parametric scanning simulation with the above geometric parameters, the results can be obtained for the resonant frequency of 6 kHz. Figure 2d,e demonstrates the effect of geometric dimensions on the maximum stresses of the devices under an input acceleration of 20 g at a frequency of 2 kHz. With the increasing of the width and thickness of the cantilever beams, the maximum stress would decrease, while the maximum stress would become larger with a longer proof mass or beam. Despite of the fluctuation of the maximum stress, the total trends of the output changes are consistent within the range of several MPa, which is far below the fatigue strength of the silicon substrate. Based on the calculation of the maximum stress and resonant frequencies, the dimensions of the cantilever beams can be determined, including the length, width and thickness of the beams and proof mass. Once the intrinsic frequency is fixed, a relatively large length of proof mass should be chosen for the sake of fabrication. Meanwhile, the thickness of the proof mass can be same as that of the cantilever beam in order to reduce the difficulty of fabrication.

As soon as we have obtained the geometric dimensions, including the cantilever beam length of 2260 μm, the cantilever beam width of 460 μm, the thickness of 60 μm, the length of a proof mass block with a square length of 3600 μm, the first-ordered to third-ordered modal analyses can be performed. Figure S2 shows resonant frequencies of 6032, 12,608 and 12,682 Hz, for the first-ordered vibration mode to the third-ordered vibration mode, respectively. Among them, a vertical displacement was found for the first-order resonant mode, whereas there would be torsional displacement in different directions for both the second- and third-order vibration modes. In order to ensure a steady performance, the working frequency of the vibration sensors should be far below the first-order resonant frequency.

### 2.3. Fabrication of the Vibration Sensor

**Figure 3:**
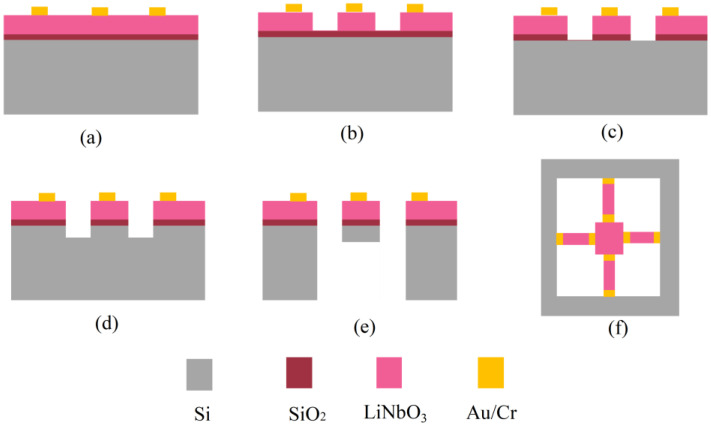
Scheme of the fabrication of the MEMS vibration sensor. (**a**) Photolithography, metal sputtering deposition and lift-off process. (**b**) LiNbO_3_ pattern produced by photolithography and IBE. (**c**) RIE etching of the SiO_2_ layer. (**d**) Cantilever beams and mass fabrication using lithography and etching by the DRIE process. (**e**,**f**) Release of the cantilever beams by the DRIE process.

Based on the results of the above analysis and consideration, the geometric parameters of the MEMS vibration sensors were determined. After this, the vibration sensor was fabricated in the standard clean room. The fabrication process for the MEMS vibration sensor is depicted in Figure 3. Briefly, a set of 5-inch chromium masks were designed and prepared according to the calculation results. Then, a 4-inch silicon wafer bonded with a thin-film single-crystalline LiNbO_3_ (X-cut, Jinan Jingzheng Electronics Co., Ltd., Jinan, China) was thoroughly cleaned prior to the photolithography and other processes. After the standard photolithography processes, including photoresist spin-coating, pre-baking, ultraviolet exposure, post-baking and development, resistive patterns could be obtained. Then, the surface electrodes could be generated by metal deposition using magnetic sputtering, as well as the lift-off process. Next, ion-beam etching (ACME POLE, Beijing, China), one of the most difficult steps, was conducted in order to achieve the patterning of the LiNbO_3_ functional structures. The IBE etching processing includes an ion-beam current of 100 mA, an etching rate of 21 nm/min, and an input of the total etching energy of 500 eV. Then, the RIE etching process was performed to obtain silica patterns. Finally, deep etching was implemented to release the cantilever beams and proof mass by the DRIE process using Omega LPX Dsi equipment (SPTS Technologies Ltd., Newport, UK), both from the front and back sides. The flow rates were 300 sccm: 400 sccm for downward etching with gas plasma of C4F8 and SF6, whereas we used a flow rate of 996 sccm for the sidewall etching with a gas plasma of SF6. The etching cycles could be manipulated to control the etching depth of the silicon precisely. During the fabrication process, common photolithography and etching processes can ensure the precise control of the dimensions of the cantilever beams and central proof mass.

### 2.4. Morphology Characterization of the Vibration Sensor

After the fabrication, the appearance of the as-prepared and packaged MEMS vibration sensor was obtained by camera photography. In addition, the detailed morphologies of the sensing parts—including the proof mass, cantilever beams, surface electrodes, and welding points, etc.—were characterized using a scanning electronic microscope (SEM, SUPRA 55, Carl Zeiss, Oberkochen, Germany). In order to investigate the cross-sectional morphology of the cantilever beam more clearly, we deliberately cut out one of the as-prepared sensors after the testing experiment. In particular, the cross-sectional view of the functional structures with LiNbO_3_ layer patterns bonded onto the silicon wafer was characterized using SEM, and the thicknesses of the three layers were measured.

### 2.5. Testing of the Performance of the Vibration Sensor

After the fabrication and packaging, a series of tests should be carried out to test the performance of the MEMS vibration sensors. This measurement is mainly composed of two parts: one is the output charge and sensitivity, and the other is the intrinsic frequency of the vibration sensor. The testing platforms can be seen in Figure S3. A MSA-400 Micro System Analyzer (Polytec China Ltd., Beijing, China) was used to test the intrinsic frequency of the vibration sensor (Figure S3a). A driving voltage of 8 V was applied with a sweep frequency ranging from 30 to 6.3 kHz. The output deflection was observed and recorded during the voltage supply, such that the intrinsic frequency could be obtained.

In order to investigate the performance of the packaged sensors, a vibration testing platform was used for the generation of vibration with frequencies ranging from 20 to 2400 Hz. For the test at room temperature, the testing sample sensor was directly fixed on the metal vibration plate (Figure S3b). The excitation input was generated as a form of sinusoidal wave from a signal generator. Meanwhile, the effect of input acceleration on the vibration sensor was studied by varying the amplitude of the acceleration from 5 to 20 g. A charge amplifier was employed to convert the output charge signal into voltage signals. An oscilloscope was used to display the output signal, and the output data could be downloaded for further analysis.

In order to explore the applicability of the as-fabricated LiNbO_3_-based MEMS vibration sensor in an extreme environment, another experimental setup was established in an enclosed chamber, where the MEMS sensor was excited with similar vibration signals to those given above, while the testing temperature was tuned from −40 to 70 °C. The experimental set-up for studying the performance of the sensor under a controlled temperature is demonstrated in Figure S4. In order to investigate the characteristics of the vibration sensor with a changing working temperature, the vibration platform should be adaptable. Thus, the sensor was first placed on a wood plate, then a water membrane, followed by a thicker metal vibration plate (Figure S4b). The input of the vibration acceleration with certain frequencies was selected in order to validate the effect of temperature on the MEMS sensor, including frequencies of 20, 50, 100, 500, 1000, 2200 and 2400 Hz.

## 3. Results and Discussion

### 3.1. Morphology of the Vibration Sensor

**Figure 4:**
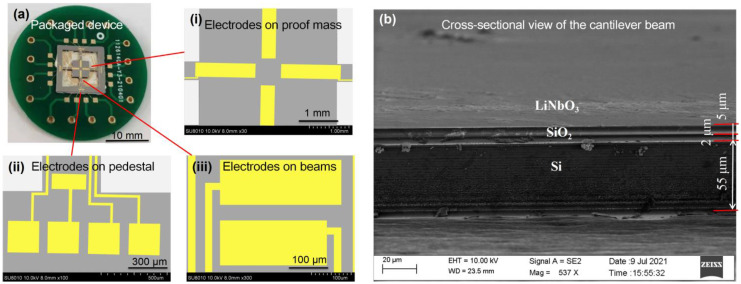
Morphological characterization of the vibration sensor. (**a**) Photograph of the packaged device with an indication of the surface electrodes on the (**i**) proof mass, (**ii**) pedestal, and (**iii**) cantilever beams, respectively. (**b**) SEM image of the cross-sectional view of the cantilever beam, indicating the layers of LiNbO_3_, SiO_2_, and silicon wafer with various thicknesses, respectively.

Figure 4 demonstrates the photography and microscopic images of the as-fabricated vibration sensor packaged on the printed circuit board (PCB). The cross-cantilever beams and center mass are clearly shown in a suspended state in the scanning electron microscope (SEM) image (Figure 4a). The metal surface electrode patterns were found intact, and they would ensure the packaging and testing of the sensor. Figure 4b shows that an etching depth of about 5 μm was achieved on the LiNbO_3_ thin film distributed on the cantilever beams, and that the slope is nearly vertical. Because the LiNbO_3_ thin film is bonded and adhered to the silicon substrate, the interface of LiNbO_3_ thin film and the silicon wafer is rather flat and clear. Moreover, the modulus of the cantilever beam is small, and it is helpful for the beam to bend about the external vibration or shock forces, so as to generate an electrical charge on different surfaces. It is the robust mechanical structure that ensures the long-term stable performance of the presented MEMS vibration sensors.

### 3.2. Performance of the Vibration Sensor

**Figure 5:**
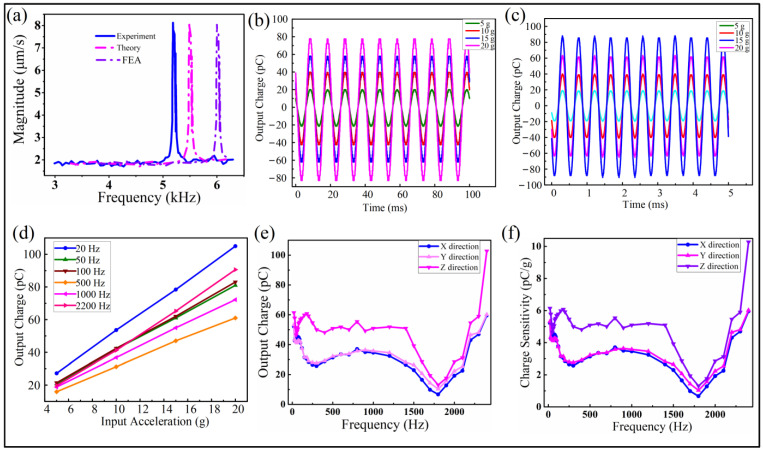
Performance of the vibration sensor. (**a**) Comparison of the intrinsic frequency obtained from the experiment, theory and FEA analysis of the vibration sensor. (**b**,**c**) Output charge performance of the vibration sensor with a sinusoidal wave with vibrations of 100 Hz and 2200 Hz, respectively, for the Z direction. (**d**) Output charge relating to the amplitude of acceleration, ranging from 5 to 20 g for the Z direction input. (**e**) Output charge for the vibration input frequencies ranging from 20 to 2400 Hz, indicating the X, Y, and Z directions. (**f**) Sensitivity of the output charge for the same range of frequencies as (**e**).

Figure 5a shows the intrinsic frequency of the vibration sensor obtained by testing. A resonant frequency of 5.175 kHz was observed for our presented MEMS vibration sensor. The measurement result agrees well with the theoretical and calculation values. However, there is still some difference between the designed target frequency 6 kHz and that of 5.48 kHz from the finite element analysis based on the Equation (1). The possible reason for the variation should be attributed to the MEMS processing, meaning that the practical geometric dimensions of the cantilever beams and proof mass are somewhat diverse compared to the ideal designed dimension. In any case, the outcome resonant frequency has proven the validation of our design of the vibration sensor.

Figure 5b,c demonstrate the output signals extracted from the oscilloscope with a perfect sinusoidal wave in testing frequencies of 100 and 2200 Hz, respectively. The results reveal the perfect response to the input vibration signals, indicating the high-performance sensitivity of the sensor. Meanwhile, by varying the amplitude of the vibration acceleration from 5 to 20 g, the output signal increased proportionally. Figure 5d shows the excellent linearity between the output charge and the input acceleration with amplitude ranging from 5 to 20 g. The slopes of the five representative frequencies are similar, indicating the similar sensitivity of the output charge response to the input acceleration.

In order to investigate the performance of the vibration sensor in a larger range of frequencies, for the potential applications, the output charge was carefully observed and recorded by scanning the input frequency ranging from 20 Hz to 2.4 kHz. Figure 5e shows the amplitude of the output charge at the same acceleration of 10 g along the Z direction, under the condition of frequency scanning. It is clear that the amplitude of the output charge increased from 61.4 to 102.8 pC. Correspondingly, the amplitude of the output charge increased from 52 to 60 pC at the same acceleration of 10 g along the X/Y direction. The output performance of the sensor during the test for 20 Hz, 1 kHz and 2.4 kHz can be also found in Figures S5–S7. For convenience, we simply obtained the sensitivity of the output charge related to the input acceleration amplitude, which was derived from Figure 5e. The results are shown in Figure 5f, indicating the output charge sensitivity increasing from 6.1 to 10.3 pC/g from 20 Hz to 2.4 kHz along the Z direction. Moreover, the output charge sensitivity would increase from 5.2 to 6 pC/g with the corresponding input frequency range along X/Y direction. The output for the Z direction vibration is higher than that of the X and Y directions. Due to the symmetry of the crossing cantilever beam structure, the X and Y directions should have a similar trend. This was verified by the comparable measurement. It was clearly shown that, for the range of frequencies between 200 to 1400 Hz, the sensitivity remained stable. However, there was a drop of output after 1400 Hz, and it reached the bottom around 1800 Hz; then, it went up again dramatically. Unfortunately, the reason for the drop in the frequencies ranging from 1400 to 2200 Hz is unknown. Considering the effect of the systematic dynamics of a mass-spring model of the presented MEMS vibration sensor, it was inferred that the air trapped around the packaged sensor might give an increased damping coefficient at that range of frequencies.

It is noteworthy that the external vibration applied to the MEMS vibration sensor comes with a certain frequency and amplitude along the vertical direction. During the vibration, the cantilever beam is deformed elastically back and forth, and subjected to the dynamic external force *F* (see Appendix A). For this mode, positive and negative charges will appear on the two electrode surfaces adhering to the LiNbO_3_ thin films along the X or Y axes, and the charge *q* can be given by:

(2) $$q=d_{ij}*F=d_{ij}*kx_{1}$$

where *d_ij_* is the piezoelectric coefficient, *k* is the effective rigidity coefficient of the vibration model, and *x*_1_ is the actual deflection of the cantilever beam due to the displacement of the proof mass. Finally, according to the analysis in the Supplementary Material, the sensitivity of the output charge of the vibration sensor *K_Q_* can be described as

(3) $$K_{Q}=\frac{q}{a}=\frac{1}{\omega_{0}^{2}}*\frac{d_{33}*k}{\sqrt{{\left(1-s^{2}\right)}^{2}+{\left(2\xi s\right)}^{2}}}$$

where m is the proof mass, ξ the effective damping coefficient, $\omega_{0}^{2}=\frac{k}{m}={\left(2\pi f_{n}\right)}^{2}$ is the intrinsic frequency of the acceleration system, and $s=\frac{\omega }{\omega_{0}}$ depicts the ratio of the input frequency to the intrinsic frequency. On basis of a theoretical analysis for the silicon cantilever beam and piezoelectric effect, the output charge and the sensitivity of the sensor can be obtained according to the piezoelectric modes.

### 3.3. Effect of the Temperature on the Performance the Vibration Sensor

**Figure 6:**
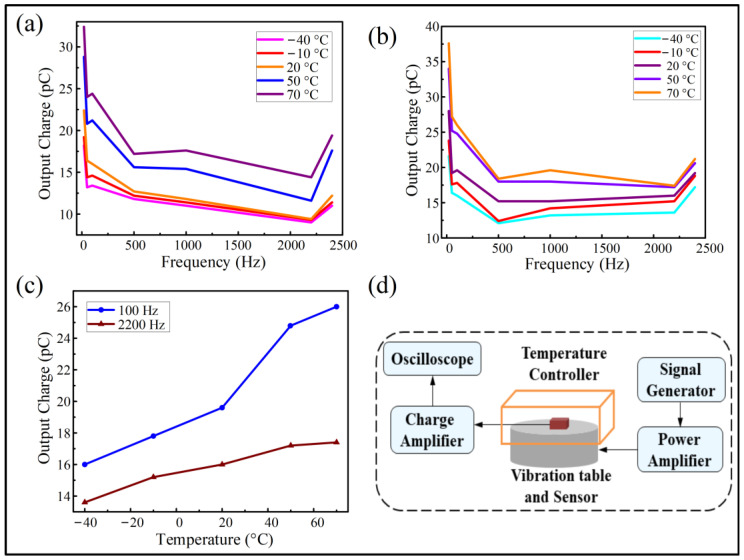
Temperature performance of the vibration sensor from −40 °C to 70 °C. Relationship of the output performance of the vibration sensor corresponding to the vibration input with frequencies from 20 to 2400 Hz, at an acceleration amplitude of 10 g, by applying vibration from the directions of the (**a**) X and (**b**) Z axes, respectively. (**c**) Curve indicating the variation of the output charge with the temperature (Z direction). (**d**) Flow chart illustrating the experimental setup for the temperature test.

In order to investigate the effect of the temperature on the performance of the sensor, a further experiment was performed at a relatively large range of temperatures. Upon reaching a stable temperature in the testing platform, a similar vibration signal was applied to the packaged sensor along different orientations of X/Y/Z, with an acceleration amplitude of 10 g. Figure 6 shows the testing results for the temperature ranging from −40 to 70 °C. Figure S5 and Figure 6d show the photo and flow chart illustrating the test platform with the control of the testing temperatures. By comparison, we emphasized the performance of the vibration sensors under the temperature conditions of −40, −10, 20, 50 and 70 °C, respectively. Figure 6a,b show the output charge performance from the application of vibrations from X direction and Z direction, respectively. It is clearly seen that the performance is quite stable for a wide-band input in the frequencies ranging from 20 to 2400 Hz.

Figure 6c indicates the variation of the output charge with the temperature from below freezing point to above the ambient temperature conditions. As shown in Figure 6c, the trend is nearly upwardly linear. It is noteworthy that the output charge slope for 100 Hz is larger than that of 2200 Hz from 20 °C to 70 °C, while they are almost the same below 20 °C (for vibration input in the Z direction). Nevertheless, for most frequencies, the variation of the output performance related to the large temperature ranging from −40 °C to 70 °C is about 5 to 7 pC for both input in X-direction and Z-direction. From the above-mentioned results, it is implied that the slight variations of the piezoelectric coefficients should be considered for the LiNbO_3_-based sensor when the temperature is changed. According to the reference [24], the piezoelectric coefficients may have an increasing trend within a certain range of temperatures. The variation can be explained with the following equation:

(4) $$\left(T\right)=d_{T_{ref}}*\left[1+k_{d}*\left(T-T_{ref}\right)\right],$$

where *d*(*T*) is the piezoelectric coefficient, $k_{d}$ is the changing rate with the temperature, $T_{ref}$ is the reference temperature (20 °C for this work), and T is the environment temperature.

Due to the differences in the platform settings, the actual input acceleration acting on the sensor should be less than that under ambient conditions, with the output charge shown in Figure 6 being only about half of that in Figure 5 for the same temperature. Nevertheless, we can obtain a similar trend of variation at a certain range of frequencies. In addition, the data acquired is comparable for the same test platform in the temperature controller.

## 4. Conclusions

In summary, in order to investigate their applicability in extreme environments, a novel-type piezoelectric MEMS vibration sensor-based LiNbO_3_ single crystal was designed to determine the geometric structural dimensions, fabricated using standard process, and tested on a vibration platform under ambient and temperature-controllable conditions. The effect of the input vibration with varying frequencies and acceleration amplitudes on the output charge was investigated. As a result, an intrinsic frequency of 5.175 kHz was observed with the presented MEMS vibration sensor, which is close to the design. During the fabrication of the vibration sensor, the patterned structure of the 5-μm-thick LiNbO_3_ layer was achieved using an ion beam etching process. The morphological characterization indicated the intact pattern and structure of the LiNbO_3_-bonded-silicon cantilever beams and the surface electrodes. The vibration sensor was tested on a standard testing platform under a vibration input with accelerations ranging from 5 g to 20 g and frequencies ranging from 20 Hz to 2.4 kHz. The results demonstrated an excellent linear trend between the output charge and the acceleration for a large range of frequencies. In the range of 1400 to 1800 Hz, the local decrease of the output charge performance might be due to the air-damping effect, with an unknown relationship. In any case, a good output charge was obtained for the wide-band vibration input, with the output charge ranging from 61.4 to 102.8 pC for the Z-direction input, and 52 to 60 pC, respectively, for the X- and Y-direction vibration inputs. In addition, the corresponding sensitivity of the output voltage increased from 6.1 to 10.2 pC/g and from 5.2 to 6 pC/g for the Z direction and X/Y directions, respectively. Moreover, the results of a wide range of temperature tests showed that there was a variation of performance. With increasing temperatures, the performance of the vibration sensor will be enhanced proportionally, indicating a change of piezoelectric coefficients with the LiNbO_3_ functional layer. Moreover, the variation of the temperature is regular and controllable. Future work will be focused on the damping effect on the performance of the vibration sensor, and for higher frequencies and lower temperatures, for practical applications in extreme environments. The presented piezoelectric-type MEMS vibration sensors—endowed with excellent linear outputs, high sensitivity to a wide-band vibration environment, and good temperature dependence—can find a potential application in extreme environment exploration.

## Figures and Tables

**Table 1:** Physical properties of the two major material settings in the numerical simulation.

Materials	Parameters	Values
Silicon layer	Density (kg/m^3^)	2330
	Young’s modulus (GPa)	190
LiNbO_3_ layer	Piezoelectric coefficients (×pC/N)	$d_{15}=68,d_{33}=6,d_{22}=22,\;d_{31}=-1$
	Density (kg/m^3^)	4700
	Young’s modulus (GPa)	240

**Table 2:** Parametric settings for the scanning simulation using Comsol Multiphysics.

Parameters for Scanning	Range of Parameter	Steps
Length of cantilever beams	1860 to 2260 μm	50 μm
Length of proof mass	3000 to 4000 μm	50 μm
Widths of cantilever beam	300 to 600 μm	20 μm
Thicknesses of cantilever beam	30 to 60 μm	5 μm
Thicknesses of proof mass	30 to 60 μm	5 μm

**Figure A1:**
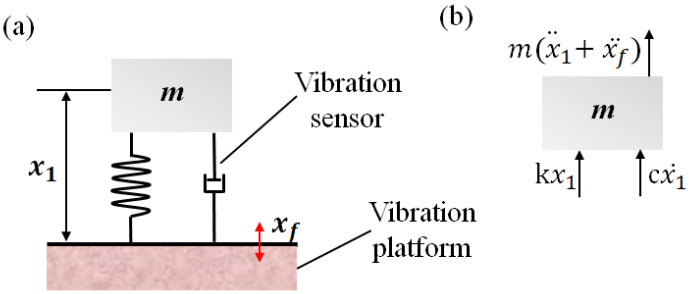
Model of the vibration system. Diagram illustration of (**a**) the vibration system model and (**b**) forces acting on the mass, according to the d Alembert principle.

## Supplementary Materials

The following supporting information can be downloaded at: https://www.mdpi.com/article/10.3390/mi13020329/s1, Figure S1: Comsol Multiphysics model indicating the substrate and pedestal, proof mass, and cantilever beams; Figure S2: Modal analysis using Comsol Multiphysics, indicating (a) the first-ordered resonance, (b) second-ordered resonance, and (c) third-ordered resonance, respectively; Figure S3: Photograph of (a) the test system and (b) the vibration platform of the MEMS vibration sensor in ambient conditions; Figure S4: Photographs of the appearance of (a) the outside and (b) the inside of the vibration platform for the testing sensor with a temperature controller; Figure S5: Signal of the output voltage at 20 Hz; Figure S6: Signal of the output voltage at 1 kHz; Figure S7: Signal of the output voltage at 2.4 kHz; Video S1: Movie displaying the output signal of the sinusoidal wave at 2.4 kHz in response to the input vibration.

## Appendix A

The output performance for the MEMS vibration sensor was tested on the vibration testing platform. Thus, the actual output of the sensor itself should be subjected to the whole vibration platform. The testing system can be seen as a single-freedom two-level damping vibration model, mainly including the vibration platform, the central proof mass, the crossing cantilever beams as the elastic component, and the interior damping due to the air resistance (Figure A1). The practical displacement of the vibration platform $x_{f}$ can be expressed as

(A1) $$x_{f}t=De^{i\omega t}$$

where *D* is the amplitude of the platform and ω is the frequency of the vibration.

According to the d Alembert principle, the vibration system dynamic equation for the above model can be described as:

(A2) $$m\left(\ddot{x_{1}}\left(t\right)+\ddot{x_{f}}\left(t\right)\right)+c\dot{x_{1}}\left(t\right)+kx_{1}\left(t\right)=0$$

where $x_{1}$ is the displacement of the proof mass related to the vibration platform, *m* is the proof mass, c is the effective damping coefficient, and k is the effective rigidity coefficient.

Substituting the second derivative of $x_{f}$, we obtain the governing equation:

(A3) $$m\ddot{x_{1}}\left(t\right)+c\dot{x_{1}}\left(t\right)+kx_{1}\left(t\right)=mD\omega^{2}e^{i\omega t}$$

Solving this governing equation, we obtain the analytic solution of $x_{1}\left(t\right),$ which is written as:

(A4) $$x_{1}\left(t\right)=\beta Be^{i\left(\omega t-\theta \right)}$$

where $mD\omega^{2}=F_{0}$, $B=\frac{F_{0}}{k}$ is the static displacement, $\omega_{0}^{2}=\frac{k}{m}={\left(2\pi f_{n}\right)}^{2}$ is the intrinsic frequency of the acceleration system, and $s=\frac{\omega }{\omega_{0}}$ is the ratio of the excitation frequency to the intrinsic frequency.

By substituting the above factors, the analytic solution of $x_{1}\left(t\right)$ can be expressed as

(A5) $$x_{1}\left(t\right)=\frac{s^{2}}{\sqrt{{\left(1-s^{2}\right)}^{2}+{\left(2\xi s\right)}^{2}}}De^{i\left(\omega t-\theta \right)}$$

where ξ is the relative damping coefficient. Thus, according to the amplitude and phase frequency distinction curves of the second-order system of a single degree of freedom, the displacement subjected to the acceleration is described as

(A6) $$\left|\frac{x_{1}\left(t\right)}{a}\right|=\frac{1}{\omega_{0}^{2}}*\frac{1}{\sqrt{{\left(1-s^{2}\right)}^{2}+{\left(2\xi s\right)}^{2}}}$$

where the lagging phase angle *θ* of the response displacement of the central proof mass related to the excitation displacement is given by

(A7) $$\theta =\tan^{-1}\frac{2\xi s}{1-s^{2}}$$

Working in the range of elastic displacement, the deformation of the cantilever beams of the vibration sensor equals the relative deflection $x_{1}$. For the inertia force *F* acting on the sensor, we obtain the expression $F=kx_{1}$. Thus, the output charge generated from the piezoelectric layer can be described as:

(A8) $$q=d_{ij}*F=d_{ij}*kx_{1}$$

Therefore, we obtain the relationship of the deflection of the cantilever beam and the acceleration, expressed as

(A9) $$\frac{x_{1}}{a}=\frac{q}{a}*\frac{1}{d_{ij}*k}$$

Finally, the sensitivity of the output charge of the vibration sensor can be described as

(A10) $$K_{Q}=\frac{q}{a}=\frac{1}{\omega_{0}^{2}}*\frac{d_{ij}*k}{\sqrt{{\left(1-s^{2}\right)}^{2}+{\left(2\xi s\right)}^{2}}}$$
