# Supplementary material for: High-Performance Piezoelectric-Type MEMS Vibration Sensor Based on LiNbO3 Single-Crystal Cantilever Beams
Source: Micromachines (Basel). 2022 Feb 19;13(2):329. doi: 10.3390/mi13020329 (PMC8879162; doi:10.3390/mi13020329)
Supplement: Supplementary file 1 [file micromachines-13-00329-s001.zip › micromachines-1587357-supplementary.pdf]

Supplementary Materials

High-Performance Piezoelectric-Type MEMS Vibration Sensor Based on $LiNbO_3$ Single-Crystal Cantilever Beams

Huifen Wei [1], Wenping Geng [1,*], Kaixi Bi [1], Tao Li [1], Xiangemeng Li [2], Xiaojun Qiao [1], Yikun Shi [3], Huiyi Zhang [1], Caiqin Zhao [1], Gang Xue [1] and Xiujian Chou [1,*]

[1]*Science and Technology on Electronic Test and Measurement Laboratory, North University of China, Taiyuan 030051, China*
[2]*Shanxi Provincial Key Laboratory of Advanced Manufacturing Technology, North University of China, Taiyuan 030051, China*
[3]*Beijing Space Trek Technology CO., LTD., Beijing 100176, China*
**Correspondence: wenpinggeng@nuc.edu.cn (W.G.); chouxiujian@nuc.edu.cn (X.C.)*

Figures and Videos:

Figure S1. Comsol Multiphysics model indicating the substrate and pedestal, proof mass, and cantilever beams.

Figure S2. Modal analysis using Comsol Multiphysics, indicating (a) the first-ordered resonance, (b) second-ordered resonance, and (c) third-ordered resonance, respectively.

Figure S3. Photo of (a) test system and (b) vibration platform of MEMS vibration sensor in ambient condition.

Figure S4. Photos of appearance (a) outside and (b) inside of the vibration platform for testing sensor with temperature controller.

Figure S5. Signal of output voltage obtained at 20 Hz.

Figure S6. Signal of output voltage obtained at 1 kHz.

Figure S7. Signal of output voltage obtained at 2.4 kHz.

Video S1. Movie displays the output signal of the sinusoidal wave at 2.4 kHz response to the input vibration.

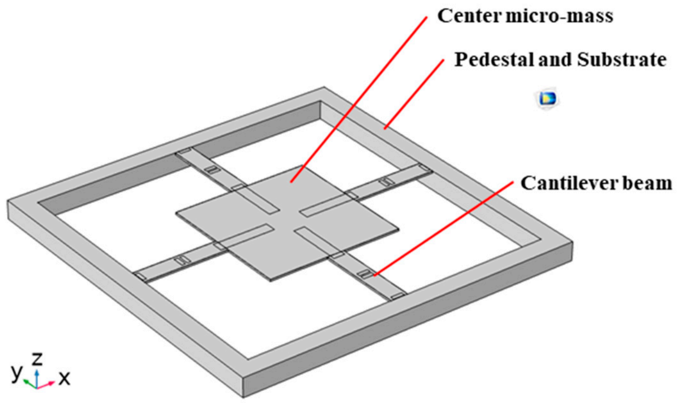

Figure S1. Comsol Multiphysics model indicating the substrate and pedestal, proof mass, and cantilever beams.

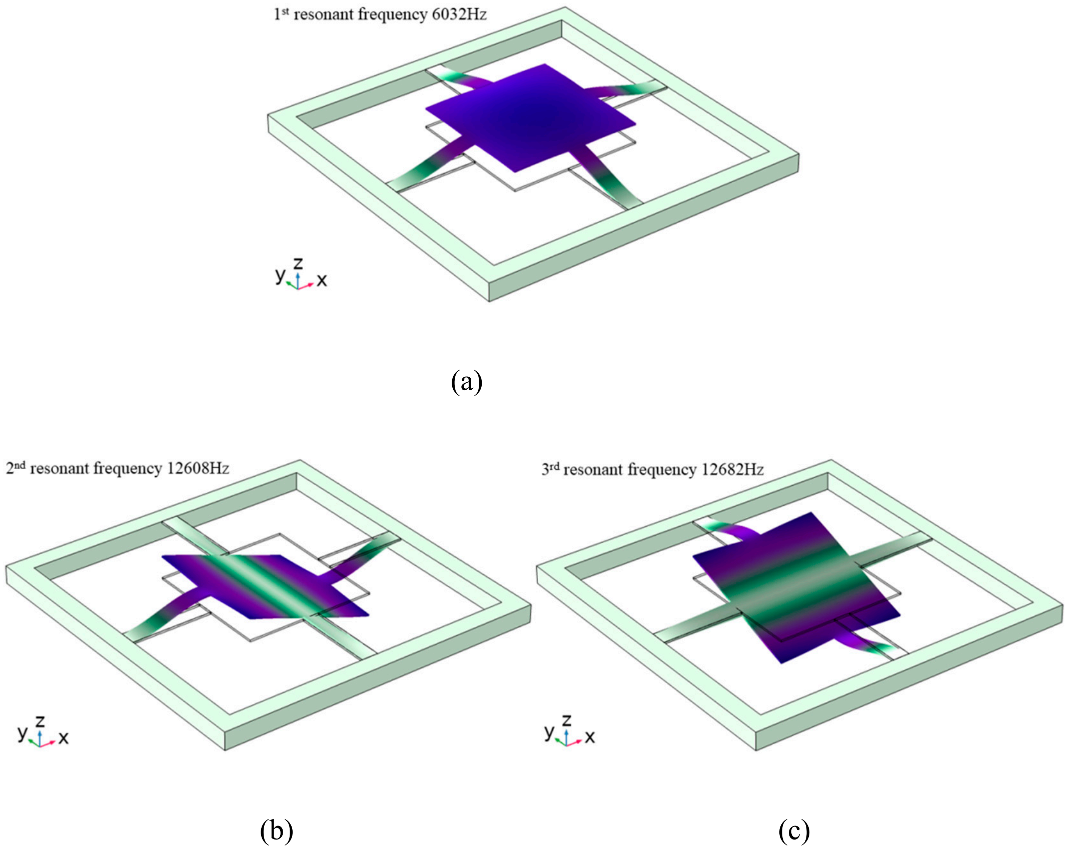

Figure S2. Modal analysis using Comsol Multiphysics, indicating (a) the first-ordered resonance, (b) second-ordered resonance, and (c) third-ordered resonance, respectively.

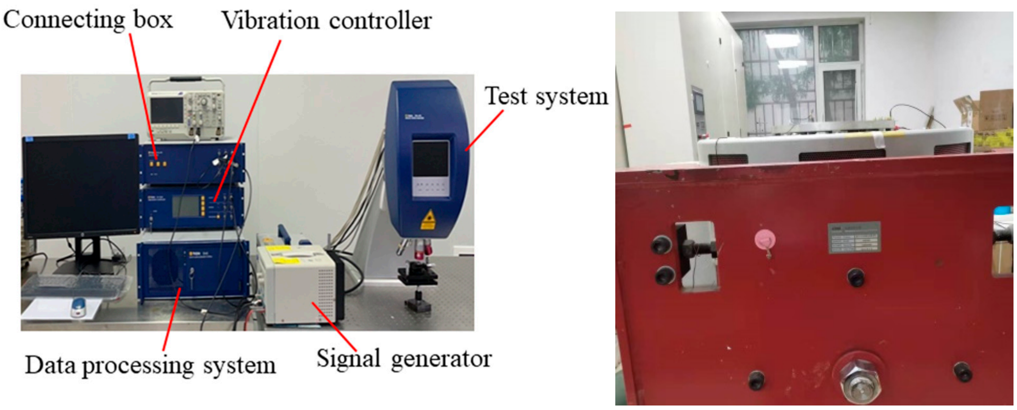

(a) (b)

Figure S3. Photo of (a) test system and (b) vibration platform of MEMS vibration sensor in ambient condition.

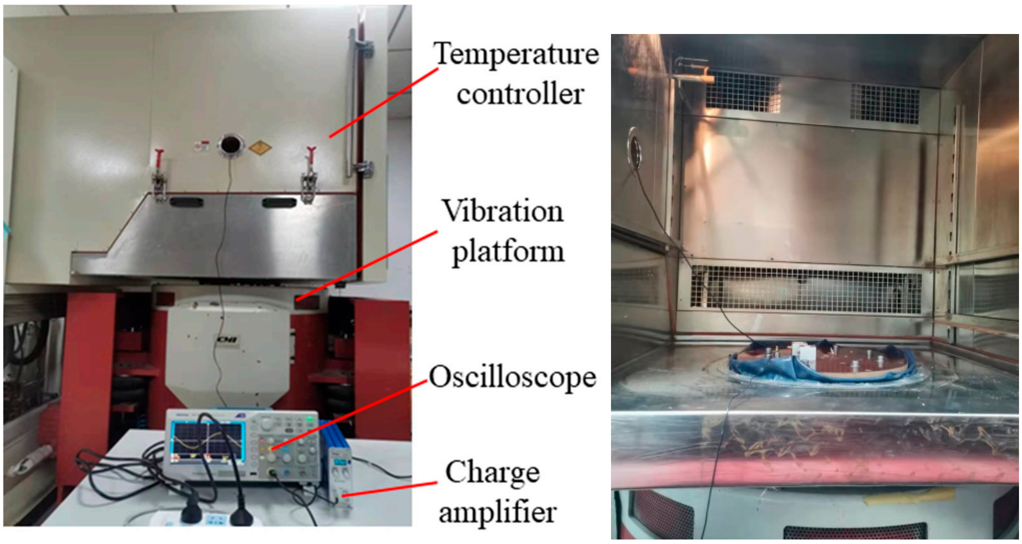

(a) (b)

Figure S4. Photos of appearance (a) outside and (b) inside of the vibration platform for testing sensor with temperature controller.

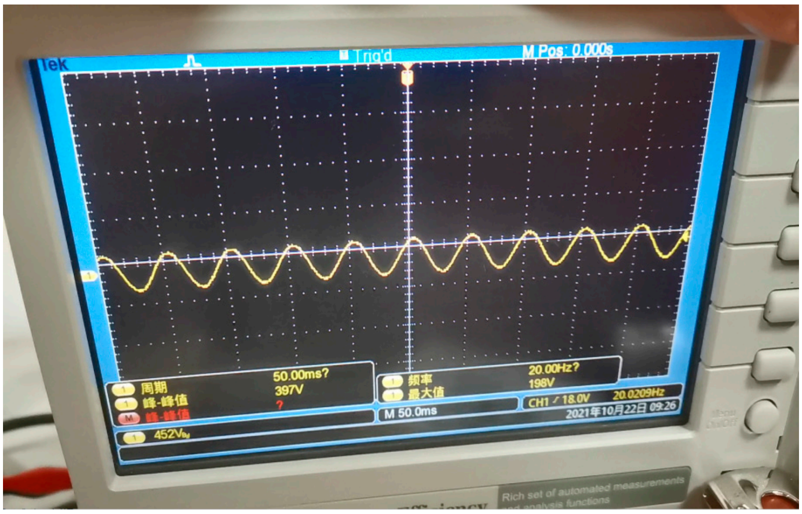

Figure S5. Signal of output voltage obtained at 20 Hz.

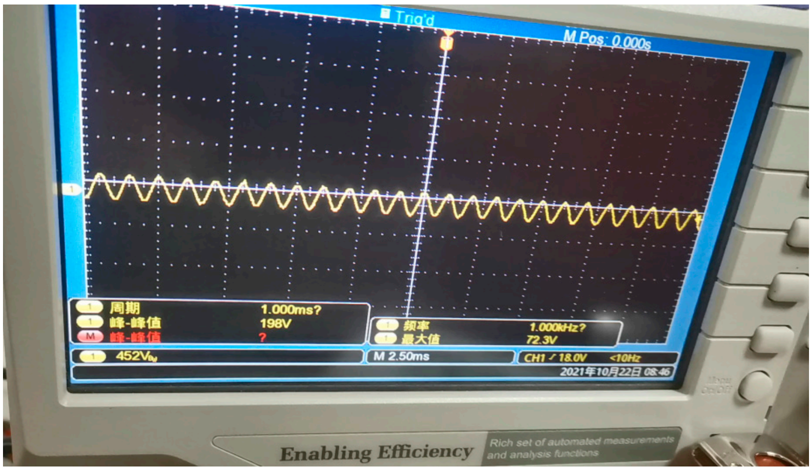

Figure S6. Signal of output voltage obtained at 1 kHz.

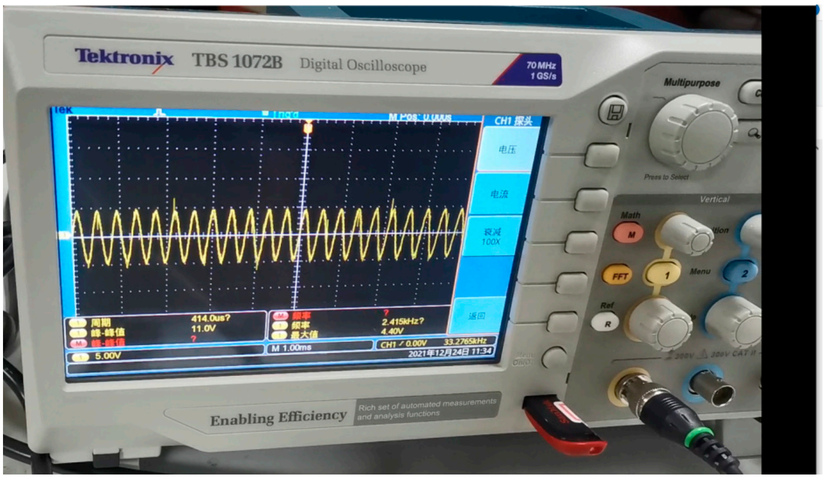

Figure S7. Signal of output voltage obtained at 2.4 kHz.
